# Physicochemical Characteristics, Fatty Acids Profile and Lipid Oxidation during Ripening of Graviera Cheese Produced with Raw and Pasteurized Milk

**DOI:** 10.3390/foods11142138

**Published:** 2022-07-19

**Authors:** Maria D. Ioannidou, Martha Maggira, Georgios Samouris

**Affiliations:** Department of Hygiene and Technology of Food of Animal Origin, Veterinary Research Institute, Hellenic Agricultural Organization-DEMETER, Campus of Thermi, 57001 Thessaloniki, Greece; ioannidou@vri.gr (M.D.I.); marthamaggira@gmail.com (M.M.)

**Keywords:** cheese, raw milk, pasteurized milk, physicochemical properties, fatty acids

## Abstract

The production of cheese can be made from either pasteurized or non-pasteurized milk, depending on the country or dietary habits. In this work, the effect of pasteurization of milk on the progress of the physicochemical properties, fatty acids profile and lipid oxidation of cheese throughout a maturation period of 90 days is presented. This research was carried out on two types of Graviera cheese produced in Greece, one made from raw milk and the other from pasteurized milk. The proximal composition of each sample was evaluated, the fatty acids profile was analyzed by Gas Chromatography, whereas lipid oxidation was determined on the basis of the formation of malondialdehyde (MDA). Significant differences (*p* < 0.05) in the values of pH, fat and density between raw and pasteurized milk were observed. The physicochemical parameters during the ripening of the cheeses showed significant differences according to the type and the stage of maturation. Specifically, the two types of cheese differed significantly (*p* < 0.05) in terms of pH, protein, fat in dry matter (FDM), and water-soluble nitrogen/total nitrogen (WSN/TN). Although the fatty acids profile was similar for the two types of cheese, differences were observed during the ripening stages as well as between the milk and the final product. The lipid oxidation levels increased during maturation, whereas they seemed to be lower in the pasteurized cheeses. Therefore, it can be concluded that the use of raw or pasteurized milk affects the physicochemical characteristics, fatty acids profile and lipid oxidation of Graviera cheese during ripening.

## 1. Introduction

Traditional manufacture of cheese from raw milk is an important feature of cheesemaking culture. The microbial diversity of raw milk contributes to the production of cheeses with unique organoleptic characteristics [1]. Because of their rich and stronger taste compared to pasteurized cheeses, raw cheeses have acquired popularity among some consumers [2]. This distinct flavor is the result of various metabolic activities that occur during the cheese’s manufacturing and maturing [3]. The fermentation of milk components by the natural microbiota of raw milk (beneficial bacteria found include *Lactococcus* spp., *Lactobacillus* spp., *Leuconostoc* spp., and *Enterococcus* spp. [2,4]) produces a substantial amount of volatile chemicals, such as carboxylic acids, esters, and alcohols, which are present in raw cheeses [5]. Pasteurization of raw milk inactivates these bacteria as well as enzymes such asproteases and lipases, which are important in improving the distinct flavor of raw cheeses [6]. Industrially produced cheeses do not have the traditional flavors, or at least lack some characteristic flavors, which is attributed to the pasteurization of milk and the use of undefined commercial starters in cheese-making [7]. As is well-known, the main goal of the milk’s pasteurization is primarily to eliminate pathogens [6]. However, pasteurization results in the type and intensity of the aromas present in milk and also leads to denaturation of the milk’s native enzymes and low-level denaturation of the whey proteins [8,9]. As a result, raw milk cheeses mature faster and have high flavor intensity; however, their flavor and taste are not always uniform [6]. Moreover, in order to produce cheeses with the same properties as raw milk cheeses, it is required to restore the beneficial microorganisms lost by heating [10]. The use of commercial starter cultures in the production of cheese from pasteurized milk results in loss of the distinctive characteristics of the same cheese made from raw milk, due to the fact that the replacement of the complex microbial flora of raw milk by a commercial culture tends to result in uniform characteristics [1].

In many countries, the production of cheese from unpasteurized milk is quite common and includes highly recognizable products (e.g., Pecorino di Filiano, Cacioricotta, Roquefort, Brie). Nevertheless, in Greece, the production of cheese based on unpasteurized milk is subjectto a very restrictive policy owing to the potential problems with safety. For example, according to the Greek regulation, the minimum ripening period should be 3 months [11]. Therefore, there are not many commercial raw cheeses and the studies in the literature for Greek raw cheeses are limited. Graviera is the finest and most popular traditional Greek cooked hard cheese [12]. The milk used for most Graviera varieties may be processed raw (thermized or pasteurized) without or with addition of natural (NSC) or commercial (CSC) starter cultures. Traditional Graviera cheese is often produced from thermized ewes’/goats’ (90:10) milk to control undesirable bacterial contaminants. Since thermization also reduces the desirable lactic acid bacteria (LAB) microbiota of raw milk, natural undefined or commercially defined starters are usually used [12]. The effect of the type of starter added to bulk thermized milk on the microbiology of mature (90-day) Graviera cheese and on gross and microbiological composition and organoleptic characteristics of Graviera Kritis cheese was evaluated [13,14].

The unique and distinct nutritional qualities of each cheese type are determined by the technological parameters utilized in cheese production. Cheeses include significant amounts of vital nutrients, such as proteins, peptides, amino acids, fat, fatty acids, vitamins, and minerals [15]. The microbiological and chemical composition of milk, cheesemaking technology, ripening period, and cheese factory conditions have a major influence on the cheese composition [16,17]. Because fat and protein are major constituents of cheese, their amounts have a significant impact on product quality. Milk fat is also a rich source of flavors, because it includes such a wide variety of fatty acids [18,19]. 

The objective of the present study was to compare the physicochemical characteristics of cheese made either from raw or pasteurized milk during ripening and to assess fatty acid profiles for both products. This was achieved by investigating parallel productions of hard, Graviera cheeses made from pasteurized and unpasteurized cow’s milk, respectively, in a Greek dairy farm. Physicochemical parameters of raw and pasteurized milk, as well as of cheese during ripening, such as pH, fat and protein content, were measured and possible statistical differences were highlighted. The fatty acid composition of raw and pasteurized cheese during ripening was determined using gas chromatography together with the lipid oxidation with spectrophotometric methods. Thus, interesting conclusions can be drawn with particular importance to the consumer public. According to our knowledge, this is the first time that such detailed physicochemical characterization, including a full fatty acid profile and lipid oxidation determination of hard, Graviera cheeses made from pasteurized and unpasteurized cow’s milk, during ripening, is presented in the literature. However, it should be stressed here that in this study only physicochemical characteristics of the products were evaluated, whereas a sensory and a microbiological analysis of the two types of cheeses would be the subject of another study.

## 2. Materials and Methods

### 2.1. Materials and Reagents

The reagents used in the analyses included: sulfuric acid (H_2_SO_4_) 98% for analysis (PanReac AppliChem, Barcelona, Spain), amylalcohol (C_5_H_11_OH) (ChemLab, Zedelgem, Belgium), sodium hydroxide NaOH (Merck, Darmstadt, Germany), boric acid (H_3_BO_3_) (PanReac AppliChem, Darmstadt, Germany), silver nitrate (AgNO_3_) 0.1 mol (Fixanal, Fluka, Munchen, Germany), ammonium thiocyanate (NH_4_SCN) 0.1 Ν (ChemLab, Zedelgem, Belgium), nitric acid (HNO_3_) 65% (ChemLab, Zedelgem, Belgium), ammonium iron (III) sulfate 12 hydrate (PanReac AppliChem, Barcelona, Spain), sodium hydroxide 0.1 mol/L (ChemLab, Zedelgem, Belgium), Tashiro’s indicator solution (Honeywell Fluka, Munchen, Germany), hydrochloric acid (HCl) 0.1 Ν (VWR Chemicals BDH, Rosny-sous-Bois cedex, France), sodium bisulfate (NaHSO_4_) anhydrous (Fluka Analytical, Munchen, Germany), sodium sulfate anhydrous (Na_2_SO_4_) (Lach-Ner, Neratovich, Czech Republic), n-hexane pesticide grade (ChemLab, Zedelgem, Belgium), tablets Kjeldahl Cu (Gerhardt, Königswinter, Germany), potassium hydroxide (KOH) 85% (Panreac, Barcelona, Spain), methanol (VWR chemicals, Radnor, PA, USA), 2-thiobarbituric acid (Sigma-Aldrich, Darmstadt, Germany), butylated hydroxytoluene (Sigma Aldrich, Darmastadt, Germany) and trichloroacetic acid (Merck, Darmstadt, Germany).

The reference standards used included: a 37 component mixture of fatty acids methyl esters, FAME mix C_6_-C_24_ (SIGMA 18919-1AMP, certified reference material), a 13 polyunsaturated fatty acids mixture, PUFA No.1 (marine source analytical standard from SIGMA, 47033 with C14:0, C16:0, C16:1 n7, C18:1 n9, C181 n7, C18:2 n6, C20:1 n9, C18:4 n3, C22:1 n11, C22:1 n9, C20:5 n3, C22:5 n3, C22:6 n3), a 14 polyunsaturated fatty acids mixture PUFA No.2 (animal source analytical standard, SIGMA 47015-U with C14:0, C16:0, C16:1 n7, C18:0, C18:1 n9, C18:1 n7, C18:2 n6, C18:3 n6, C18:3 n3 C20:1 n9, C20:2 n6, C20:3 n6, C20:4 n6, C20:5 n3, C22:4 n6, C22:5 n3 C22:6 n3) and a linoleic acid, conjugated methyl ester standard (CLA), purchased from Sigma Aldrich (SIGMA 05632) (St Louis, MO, USA). CLA included a mixture of cis- and trans-9,11- and -10,12-octadecadienoic acid methyl esters with cis-9,trans-11 being the major isomers.

### 2.2. Cheesemaking

Two batches of Graviera cheese were produced, each one of 200 L, as follows: raw milk was warmed at 35 °C and the quantity of rennet (Bioren, Italia) required curdling the milk within 30–35 min was added. No defined starter cultures were added to this batch (batch-R). It should be pointed out here that since the microbiota of raw milk consists of a large number of beneficial bacteria, the cheesemaking can take place with their own microflora. Thus, we did not consider it necessary to use a starter culture. At the same time, another batch was produced with heat treated cow’s milk (batch-P). Pasteurized (65 °C, 30 min) cow’s milk was warmed at 40 °C and a starter culture was added (mesophilic lactic acid bacteria consisting of strains of *Lactocucus Lactis*, *Leuconostoc mesenteroides*, *Streptococcus thermophilus* and *Lactobacillus helveticus*). After 30 min, the milk was cooled to 35–36 °C for the addition of CaCl_2_ (0.2%) and the rennet. After about 35–40 min from the addition of the rennet, the milk of both batches (R and P) was coagulated. The curd was cut (at pH 6.3–6.4) into small pieces (1 cm × 1 cm × 1 cm in size) and cooked for 20–30 min at 50–52 °C with continuous stirring. It was then placed in molds and pressed for 1–2 h (1–2 times their weight) before being inverted for molding and drainage of the cheese. After 24 h and when the pH of the cheese was 5.4, it was transferred into brine (18–20 Be) at a temperature of 13–14 °C. For each kg of cheese, 8–9 h of staying in the above mentioned brine was needed. The cheeses were transferred to the ripening (for 10–15 days at 13–14 °C, relative humidity 78 ± 3%), with inversions every 1–2 days. After the end of the first ripening period, the cheeses were air-sealed closed and kept at a lower temperature (6–8 °C) for at least 3 months to continue ripening (second ripening period). For each batch, samples of milk and cheese were taken after 1, 7, 15, 30, 60 and 90 days. The cheesemaking trials were carried out five times in total.

### 2.3. Physicochemical Analysis

The proximal composition (fat, protein, lactose, minerals, non-fat solids and freezing point) and the density of each sample of milk was evaluated using a FUNKE GERBER LactoStar Dairy Analyser (Berlin, Germany). Milk pH was measured with an electronic Consort pH-meter (Turnhout, Belgium).

Cheese samples were analyzed for fat, protein, pH, NaCl, moisture, water-soluble nitrogen and lipid oxidation. The moisture content of cheese samples (2–5 g) was determined after drying at 105 °C until constant weight [20]. Results were expressed as a percentage (%). The fat content of grated cheese samples was determined according to the Gerber–Van Gulik method [21]. The determination of fat in dry matter (FDM), was calculated using the obtained values of fat, Fat (%), and moisture, Moisture (%), using Equation (1):

(1) $$\mathrm{FDM}=\frac{\mathrm{Fat}\;\left(\%\right)}{\left(100-\mathrm{Moisture}\;\left(\%\right)\right)}$$

Total Nitrogen (TN) of cheese samples was measured by the Kjeldahl method [22] and the protein content was estimated by multiplying TN by a factor of 6.38. Water-Soluble nitrogen (WSN) was also determined by the Kjeldahl method according to the International Dairy Federation (IDF) [23]. The ratio of WSN⁄TN (%) was termed as ripening index. The modified Volhard test [24] was used to determine the NaCl content, and the salt-in-moisture (S/M) content was estimated. Duplicate analysis was performed for all parameters in consideration.

### 2.4. Fatty Acids Analysis

The fatty acid methyl esters were prepared by transesterification with potassium hydroxide according to ISO 5509:2000 slightly modified by Zeppa et al. [25]. Briefly, initially 10 g of cheese were homogenized. Following, an aliquot (0.15 g) was collected in a screw-capped test tube and Na_2_SO_4_ anhydrous (1 g), n-hexane (4 mL) and methanolic KOH solution (300 μL; 2 mol L^−1^) were added. The test tube was closed and shaken vigorously for about 1 min. After adding 1.5 g of NaHSO_4_ monohydrate the tube was shaken again for 30 s to neutralize the excess of KOH. The organic upper layer containing methyl esters was then transferred into a vial and immediately analyzed. Fat from milk samples was separated by centrifuging 50 mL of fresh milk for 15 min. Milk fat was methylated according to the previous procedure [25].

The fatty acid methyl esters were analyzed using an HP 6890 (Hewlett-Packard) gas chromatograph equipped with a split/splitless injector (split mode) and a flame ionization detector (FID). Samples were introduced onto a DB-23 (60 m × 0.25 mm ×0.25 µ m) column (J & W Scientific, Inc., Folsom, CA, USA) via 1-μL split injections with a split ratio of 50:1. Automated split injection was performed using a Hewlett-Packard 7683 autosampler. Injection took place at 250 °C and FID temperature was set at 280 °C. The oven temperature program used was: hold for 1 min at 40 °C; heating from 40 °C to 175 °C at a rate of 25 °C min^−1^; heating from 175 °C to 250 °C at a rate of 4 °C min^−1^; hold for 10 min at 250 °C. Column head pressure was set at 32.44 psi (constant pressure). A split injection liner (4 mm i.d.; Agilent, CA, USA) was used for all injections. Gas flow rates were as follows: helium (carrier), 2.1 mL min^−1^; helium (make up), 30.0 mL min^−1^; compressed air, 350 mL min^−1^; and hydrogen, 45 mL min^−1^.

Fatty acids methyl esters were identified by comparing their retention times with the retention times of the reference standards and the results are expressed as percent (%) of the total fatty acids present in the sample.

From the percentages of the fatty acids *C*12:0, *C*14:0 and *C*16:0, the Atherogenicity Index (AI) was calculated according to the following equation:

(2) $$\mathrm{AI}=\frac{C12:0+4\times C14:0+C16:0}{\left(\mathrm{PUFA}+\mathrm{MUFA}\right)}$$

where PUFA and MUFA denote the polyunsaturated and monounsaturated fatty acids, respectively.

### 2.5. Lipid Oxidation

Lipid oxidation was determined on the basis of the formation of malondialdehyde (MDA) using a selective third-order derivative spectrophotometric method [26]. Samples were homogenized in an IKA homogenizer (Ultra Turrax, Staufen, Germany). Two grams of sample were homogenized with aqueous trichloroacetic acid (TCA) in the presence of hexane containing butylated hydroxytoluene (BHT) and the mixture was centrifuged. After centrifugation, the top hexane layer was discarded. The bottom aqueous layer was increased to 10 mL of volume with TCA, and a 2.5 mL aliquot was pipetted into a screw-capped tube in which a volume of 0.8% aqueous TBA was also added. The mixture was incubated for 30 min at 70 °C. The mixture was cooled in a cold-water bath after incubation, and the absorbance was measured at 521.5 nm against a blank sample using a Shimadzu model UV-1601 spectrophotometer (Tokyo, Japan). The concentration of MDA in analyzed samples was calculated on the basis of the height of the third order derivative peak at 521.5 nm by referring to the slope and intercept data of the computed least squares fit of a standard calibration curve prepared using 1,1,3,3-tetraethoxypropane. Lipid oxidation is expressed as nanograms of MDA per gram of cheese.

### 2.6. Statistical Analysis

Comparison of the physicochemical properties between raw and pasteurized milk were assessed by the Student’s *t*-test (5% level of significance). A one-way analysis of variance (ANOVA), using the Tukey test for pair wise comparison of means, was performed (significant level of 5%) to follow the changes in the physicochemical parameters and fatty acids profile during cheese ripening. In cases where the distribution was not normal, the comparisons were made with the non-parametric tests Kruskal–Wallis and Mann–Whitney. Pearson’s correlation coefficients were used to determine the association between certain variables. The statistical analysis was performed using the statistical software Jeffreys’s Amazing Statistics Program JASP (JASP v 0.14. https://jasp-stats.org/download/ (accessed on 20 January 2020).

## 3. Results and Discussion

### 3.1. Physicochemical Characteristics of Milk

The physicochemical characteristics of the milk used for manufacturing the cheese are reported in Table 1. Among the analyzed parameters, a significant difference was only found between the values of pH, fat and milk’s density (*p* < 0.05). A lower fat content of the pasteurized milk compared to raw milk has been also reported in the literature [27,28]. However, the reduction observed in this investigation was not that great, although statistically significantly different (seen by the different superscripts in Table 1) due to the relatively low pasteurization temperature used. As was reported previously, pasteurization took place at 65 °C for 30 min. According to the International Dairy Foods association, (IDFA) pasteurization at 145 °F (63 °C) for 30 min is defined as Vat Pasteurization [29] and was the original method of pasteurization. Nowadays, it is known as Low Temperature Long Time (LTLT) treatment and it still is one of the most commonly used methods for pasteurization of fluid foods and raw materials [30]. Although the most common method of pasteurization today is High Temperature Short Time (HTST), we prefer to use the low temperature technique in order to not significantly affect the physicochemical parameters of the raw milk, as indeed was the case.

### 3.2. Physicochemical Characteristics of Cheese

It has been found in the literature that pasteurization and ripening temperature has a significant effect on pH, acidity, dry matter, fat, protein and salt (*p* < 0.05), which were higher in pasteurized cheeses ripened at a higher temperature (12 °C) [31].

The measured physicochemical properties of the two types of Graviera cheese (raw and pasteurized) over the ripening period are exhibited in Table 2. The statistical analysis of the physicochemical characteristics revealed significant differences (*p* < 0.05) depending on ripening stage and cheese type.

pH values showed no significant (*p* > 0.05) changes over time in both samples. pH showed a decrease in the first 30 days for the raw cheese and in the first 15 days for pasteurized one due to fermentation of lactose to lactic acid, as well as the liberation of free fatty and amino acid by proteolysis and lipolysis [32]. The comparison of the pH values between the two types of cheese showed a lower pH value for raw milk cheeses compared to the pasteurized one (*p* < 0.05), which is consistent with previous studies [1,32,33,34]. This may be due to the elimination of the native enzymes and microflora (which are responsible for the pH decrease in cheese during ripening) by heat treatment during pasteurization.

The cheese moisture gradually decreased over time presenting a significant difference between the 1st and the 90th day of ripening for both cheese types. This is attributed to the dehydration caused by water loss and volatile component exchanges between the cheese surface and the ripening room environment (fat, volatile fatty acids, etc.) [35]. According to the moisture data in 90 days of ripening, both types of cheese have an average moisture equal to 35%, which categorized them as hard cheeses.

The fat content and the FDM increased slightly over ripening time, without significant differences, for both raw and pasteurized cheese. This could be due to an increase in dry matter when the cheese is placed in the brine, which occurs in parallel with a decrease in moisture content that may contribute to an increase in the fat content. The results were similar with others reported for similar-type Greek cheeses [36]. Comparison of the two types of cheeses indicated higher FDM values for pasteurized cheese statistically different (*p* <0.05) from the raw cheese. Thus, it was verified that pasteurization increases the fat in dry matter. 

The NaCl (%) increased in both types of cheese during ripening, attributed to the diffusion of NaCl molecules from the brine to the cheese body. There were statistically significant variations between the stages of ripening. However, no significant differences between the two types of cheeses were observed. The Salt in Moisture (S/M) parameter follows a similar course. The profile of salt-in-moisture concentration versus ripening time can be explained by both the diffusion of dry salt from the surface into the center of the cheese, where it gives rise to an increased salt content and by the water evaporation from the surface of the cheese, which accounts for the decreased moisture content. The NaCl content was 1.8% for both cheese types, which agrees with the findings of another study for hard cheeses [36].

Proteolysis is the most important phenomenon during ripening, which determines both texture and flavor development in cheese. Proteins are partially hydrolyzed by rennet, and other native or microbial enzymes, to produce lower molecular weight compounds, and are further broken down by peptidases into various nitrogenous substances, such as proteose, peptone, amino acids and amines [9,19]. It has been proposed thatthe application of high hydrostatic pressure treatment significantly accelerates the proteolysis during ripening [37,38].

According to Table 2, the protein content slightly increased from an average value of 25.7% during manufacture to an average of 26.6% after 90 days for the raw cheese. The protein levels for the pasteurized cheese showed a higher increase from an average value of 23.1% during manufacture to an average of 27.5% after 90 days with no significant differences between the stages of ripening.

The water-soluble Nitrogen (WSN) fraction is a very heterogeneous solution in terms of components, which include whey proteins, high-, medium- and low molecular-weight peptides and free amino acids [23]. WSN consists mainly of nitrogen-based compounds, which are produced through reactions involving rennet and milk proteinases; this network of reactions is referred to as secondary proteolysis [39]. WSN/TN has been used by many authors as a cheese ripening indicator in order to estimate the degree of proteolysis [40].

The amounts of both WSN and WSN/TN were found to be significantly higher in raw milk cheese compared to pasteurized milk cheese, indicating that the microbial load and plasmin activity were higher in raw milk [41,42]. Pasteurization destroys most of these microorganisms (non-starter lactic bacteria), and thus, delays biochemical transformations [41]. Experiments done by Aly and Gala [43] found a similar trend in WSN/TN values, which could be related to the influence of pasteurization on SN components and the existence of microbial flora and natural enzymes in raw milk and non-starter lactic bacteria [33]. It has also been found in the literature that higher ripening temperatures increase the proteolytic activity of rennet, plasmin and bacteria, which result in an increase in the WSN/TN content [44]. An increase in the pasteurization temperature from 65 to 75 °C was found to increase the WSN/TN content. However, when the temperature increased to 85 °C, the WSN/TN content was reduced [28].

Various WSN and WSN/TN contents have been reported in different cheese types. Among them, St. Paulin cheese made from raw milk was reported to contain higher amounts of WSN than pasteurized milk cheese; reference [45] determined that Canestrato Pugliese cheese made from raw milk contained 30% higher WSN than pasteurized milk cheese. However, it was reported that pasteurization of milk did not affect WSN contents of Cheddar [9,19] and Swiss-type cheeses [41].

**Figure 1:**
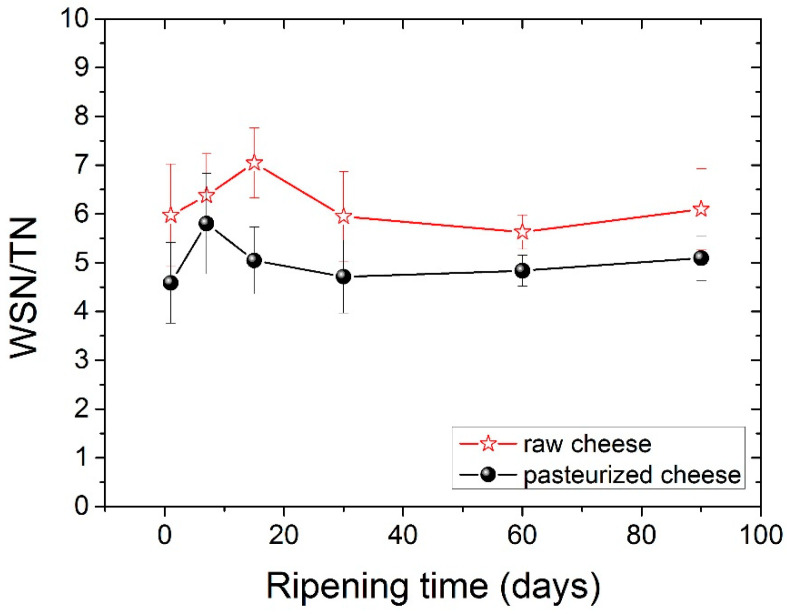
Ripening index (WSN/TN) for both types of cheese during ripening.

In this study, the WSN/TN ratio slightly increased during ripening with no significant differences (*p* < 0.05) among ripening stages. The comparison of the WSN/TN values between two types of cheese showed a higher WSN/TN value for raw milk cheese compared with the pasteurized one (*p* < 0.05), consistent with previous studies [46], and shows that maturation occurs to agreater extentin raw cheese. The values of the WSN/TN ratio during ripening for both types of cheese are shown clearly in Figure 1.

#### Correlation between Physicochemical Characteristics

To assess if there is correlation among the physicochemical characteristics, a linear correlation test was performed to raw and pasteurized cheeses (Table 3 and Table 4, respectively). In both cheeses, a negative correlation between the values of fat and moisture was observed. The negative correlation of moisture and fat seems logic as the dehydration of cheese affects the content of fat during the ripening process, since as moisture decreases, the fat increases [35]. For the raw cheese, pH value showed a strong positive correlation for moisture coordinate and negative correlation for fat coordinate. The decrease in pH is related to the action of lactic acid bacteria and the production of lactic acid associated with ripening. However, ripening is associated with a reduction in moisture. A reduction in the moisture is accompanied by an increase in the fat content. NaCl value seems to have positive correlation with protein content and strong negative correlation with WSN/TN. As for the pasteurized cheese, protein is correlated negatively with moisture and FDM values and negative with WSN/TN coordinate. The S/M coordinate presented strong positive correlation with the fat content of the cheese.

A main change in the two types of cheese that induced by pasteurization is the decrease of the pH. The comparison of the pH values between the two types of cheese showed a lower pH value for raw milk cheeses compared to the pasteurized one, which is consistent with previous studies. This may be due to the elimination of the native enzymes and microflora (which are responsible for pH decrease in cheese during ripening) by heat treatment during pasteurization. Pasteurization also induced the different protein content a fact that led to differences between WSN/TN values. The comparison of the WSN/TN values between two types of cheese showed a higher WSN/TN value for raw milk cheese compared with the pasteurized one which shows that maturation occurs to agreater extentin raw cheese.

### 3.3. Fatty Acids Profile

Cheese is more than just a source of essential compounds (calcium, protein and vitamins); it also contains a variety of bioactive molecules, the most important of which are fatty acids. Cheese fat has been shown to include about 400 different FAs, making it the most complex fat in the human diet [47]. The fatty acid (FA) profile of raw and pasteurized cheese during ripening is shown in Table 5. Concerning FAs of either raw or pasteurized cheeses, significant differences were notedfor 12 FAs out of the 42 analyzed during ripening. However, pasteurization did not have a significant effect on the fatty acid profile of cheese samples. Therefore, there is no need to use letters to indicate statistical differences between cheeses obtained from raw or pasteurized milk.

**Figure 2:**
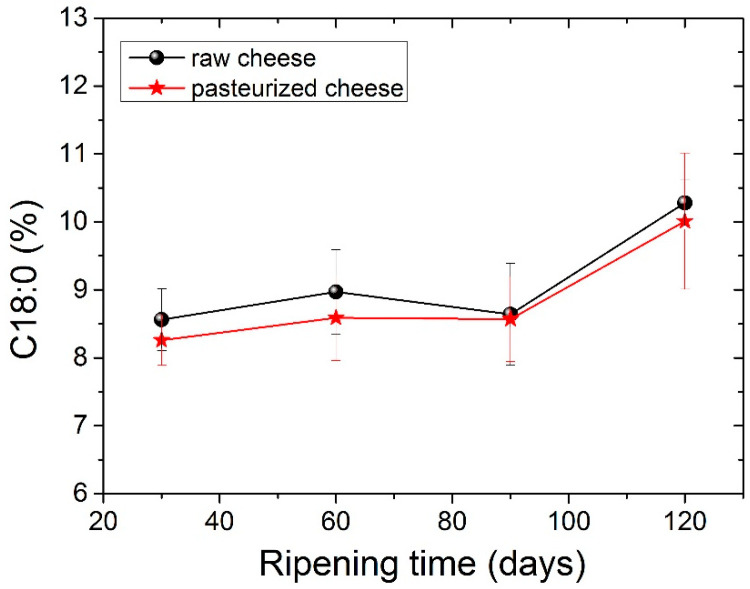
Stearic acid C18:0 values (%) for both types of cheese during ripening.

**Figure 3:**
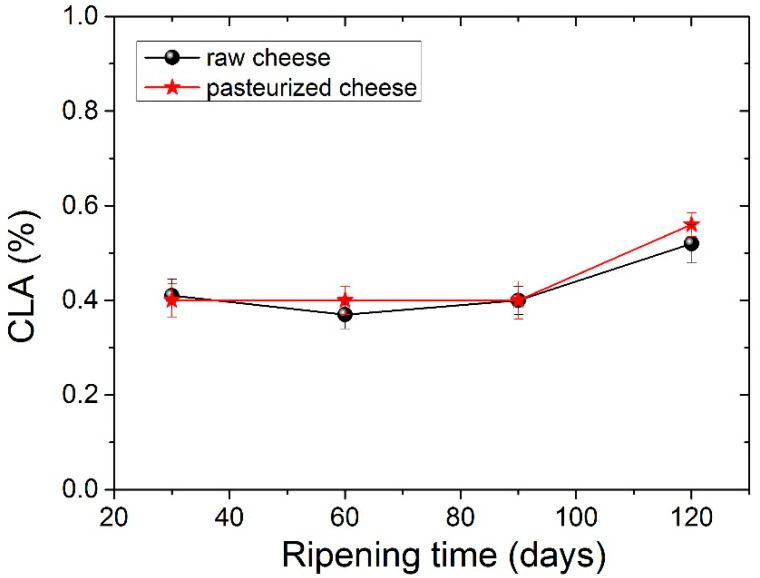
Conjugated Linoleic Acid-CLA values (%) for both types of cheese during ripening.

The four dominant FAs in both types of cheese were C14:0, C16:0, C18:0 and C18:1 c, with C16:0 (palmitic acid) being present in higher levels. The amount of palmitic acid decreased significantly (*p* <0.05) from 35.23% to 32.73% for raw cheese and 35.06% to 33.80% for pasteurized cheese after 120 days of ripening. It should be noticed here that, in all tables before Table 5, measurements for periods longer than 90 days were not carried out, since in 90 days, the cheese is considered ripened. In Table 5, we analyzed the FA of the cheese for a longer period to examine possible further differences. The C16:0 acid was also abundant in other cheeses. Particularly C16:0 dominated in Apuseni cheese (a hard cheese from cow’s milk), which also decreased during ripening [48]. It was also found in other cheeses made from cow’s milk, such as Parmigiano Reggiano cheese [49], Blue cheese, Swiss cheese, Roquefort [50] and also in many Greek varieties, such as Kasseri, Graviera [36] and Feta [51,52]. The second most abundant FA was oleic acid (18:1 n9), with an average content of ~22% in both types of cheese that were found to be stable during the ripening. Myristic acid (C14:0) was also found in large quantities, with an average content of ~11.5% in both types of cheese. Myristic acid was followed by stearic acid (C18:0), which compared to palmitic acid increases significantly (*p* < 0.05) during ripening from 8.56% to 10.28% for raw cheese and 8.26% to 10.01% for pasteurized cheese. In Figure 2 presents the percentage of stearic acid during ripening for both types of cheese. Dodecanoic (lauric) acid (C12:0) had an average value of ~3.8% in both cheeses that stayed stable during ripening. Butyric (C4:0) and capric acid (C10:0) ranged between an average content of ~2.2% and 3%, respectively, for both types of cheese. Both FAs had a slight increase during ripening and a decrease in 120 days with no significant differences; nevertheless, C4:0 showed an increased average value in milk. According to the literature, this finding indicates lipolytic activity resulting from both the starter microorganisms, and the rennet added tomilk during the cheesemaking process [52,53,54]. The amount of C18:2 c, known for the antiatherogenic action [55], increases significantly (*p*< 0.05) for both kinds of cheese from 90 to 120 days of ripening. In addition, the levels of the other FAs were low in both cheese samples in a content below 2%. Conjugated Linoleic Acid (CLA) increased significantly (*p* < 0.05) from day 90 to day 120 of ripening and had an average content of 0.52% for raw cheese and 0.56% for the pasteurized one in the final stage of ripening. The percentage of CLA during ripening it is shown in Figure 3. Studies have shown that CLA, particularly its primary isomer, cis-9, trans-11 C18:2, provides a variety of health benefits, including anticarcinogenic, antiatherogenic, immune system enhancement and antidiabetic properties [56,57].

From the values reported in Table 5, the Atherogenicity Index (AI) was calculated according to Equation (2). AI indicates the relationship between the sum of the main saturated fatty acids and that of the main classes of unsaturated fatty acids, the former being considered pro-atherogenic (favoring the adhesion of lipids to cells of the immunological and circulatory system) and the latter antiatherogenic (inhibiting the aggregation of plaque and diminishing the levels of esterified fatty acid, cholesterol, and phospholipids, thereby preventing the appearance of micro- and macrocoronary diseases) [55]. AI takes into account the different effects that single fatty acid might have on human health and in particular on the probability of increasing the incidence of pathogenic phenomena, such as atheroma and/or thrombus formation. Examining the values reported in Table 5, it seems that the AI decreases significantly between 30 and 120 days for raw cheese, whereas no statistical difference with ripening time was measured for pasteurized cheese. From Table 6, it can be evidenced that the atherogenic index is similar (not statistically different) in milk and final products, i.e., raw and pasteurized cheese. 

The GC analysis showed that most of the fatty acids were saturated (SFAs), with an average value of ~69% for both types of cheese, which is in agreement with findings of other studies [36,58]. The amount of poly-unsaturated fatty acids (PUFAs) had a slight increase, with no significant differences during ripening. The average PUFAs in the final product was 4.07% for the raw one and 4.53% for the pasteurized one. Mono-unsaturated fatty acids (MUFAs) did not change during ripening and had an average value of 26.5%. It has been observed that MUFAs are beneficial for human health, reducing the risk of coronary heart disease and lowering the blood cholesterol [55]. n-6 FAs seemed to increase significantly (*p* < 0.05) in pasteurized milk during ripening while in n-3 FAs has been observed an increase between 30 and 90 days only in raw cheese. The PUFA/SFA ratio seemed to be similar for both types of cheese.

The comparison between FAs of the raw milk that constituted the cheese and the final products (raw and pasteurized) is presented in Table 6. The FA profile of the milk was very similar to the cheese’s profile. However, statistical differences were observed in more fatty acids between milk and both types of cheeses. Specifically, the four dominant FAs in milk were the same as incheese, i.e., C14:0, C16:0, C18:0 and C18:1 c, while C16:0 (palmitic acid) was the most abundant. Myristic acid (C14:0) seemed to be present in a significantly larger amount in milk (*p* < 0.05) compared to pasteurized cheese. C4:0 and C18:2 c seemed to follow a similar course being in higher amount in raw milk. Unlike previous fatty acids, C15:0 seemed to be in significant greater amount in the final products. Moreover, differences were observed in FAs that were present in low amounts, such as C18:1 t, C18:1 c, C18:1 n7, C18:2 t, C18:3γ, C20:0, C20:3 n6, C20:4 n6, C22:2 and C22:4 n6.

Meanwhile, the amount of PUFA’s and n-6 FAs was significantly higher in milk (*p* < 0.05), and it is noteworthy that the ratio between n-6/n-3 differed significant between the milk and the final product. According to the above, it seems that many FAs are decreased during cheesemaking.

### 3.4. Lipid Oxidation

**Figure 4:**
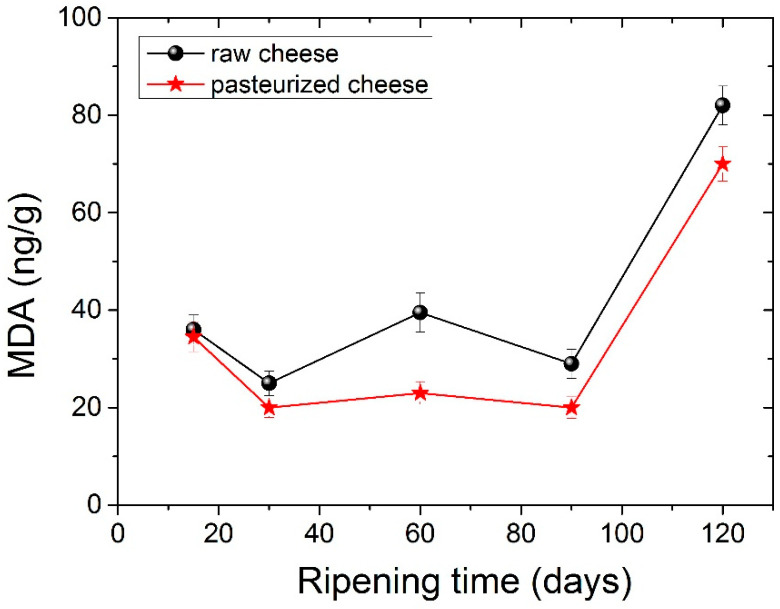
Lipid oxidation (mg MDA g^−1^) changes during ripening for raw cheese and pasteurized cheese.

Lipid oxidation in cheese samples during maturation is presented in Figure 4. Lipid oxidation values seem to increase in the second month, whereas at 90 days, they showed a slight decrease. After 120 days of ripening lipid oxidation, values were statistically significantly different compared to the corresponding values observed during the first 30 days. In general, low amounts of MDA were obtained, a fact that is in agreement with other studies [59]. Cheese from pasteurized milk seemed to have lower amounts of MDA compared to raw cheese. Statistically different values between raw and pasteurized cheese were measured at 120 days of ripening. Lipid oxidation is a significant quality issue in processed dairy products, particularly during storage. Our findings reveal that lipid oxidation does not occur to a great extent during ripening.

## 4. Conclusions

In this investigation, detailed physicochemical characterization, together with a full fatty acid profile and lipid oxidation determination of traditional Greek, hard, Graviera cheeses made from pasteurized or unpasteurized cow’s milk, during ripening, was presented.Initially, physicochemical parameters of raw and pasteurized milk, such as pH, density, fat and protein content, were measured, and statistical differences were highlighted. Next, the variation of these properties, together with the nitrogen content, moisture, NaCl and fat in dry matter of cheese during ripening, were evaluated.

Several physicochemical properties of raw and pasteurized cheese were found to differ considerably. A lower pH value for raw milk cheeses compared to the pasteurized milk cheeses was measured, which could be due to the elimination of the native enzymes and microflora (whichare responsible for a pH decrease in cheese during ripening) by heat treatment during pasteurization. Raw cheese had higher values in protein content, which is an indication of faster ripening, and therefore, impacts the cheese quality. The amount of WSN/TN was found to be higher in raw milk cheese compared to pasteurized milk cheese. FDM values also differed significantly for the two types of cheese. The atherogenic index was found to be similar in milk and final products, raw and pasteurized cheese. Moreover, no statistical difference in AI values with ripening time was measured for pasteurized cheese. However, AI decreased significantly during ripening between 30 and 120 days for raw cheese.

The fatty acid profile was similar for the two cheeses, although differences were observed between the ripening stages. Lipid oxidation did not occur to a great extent for both types of cheese, although raw cheese seemed to have higher amounts of MDA. The current study shows that dairy products such as cheese made exclusively from raw milk can be an alternative development tool on the Greek market.

## Figures and Tables

**Table 1:** Physicochemical composition of milk.

Physicochemical Parameter	Raw Milk	Pasteurized Milk
pH	6.63 ± 0.07 ^a^	6.55 ± 0.04 ^b^
Fat (%)	3.63 ± 0.49 ^a^	3.24 ± 0.08 ^b^
Solids (%)	8.64 ± 0.23 ^a^	8.81 ± 0.20 ^a^
Protein (%)	3.35 ± 0.09 ^a^	3.43 ± 0.08 ^a^
Lactose (%)	4.73 ± 0.14 ^a^	4.84 ± 0.12 ^a^
Minerals (%)	0.72 ± 0.04 ^a^	0.70 ± 0.02 ^a^
Density	1.030 ± 0.001 ^a^	1.032 ± 0.001 ^b^
Freezing point (°C)	−0.495 ± 0.014 ^a^	−0.491 ± 0.009 ^a^

Results in the same row for the same parameter with different superscripts are significantly different at *p* < 0.05.

**Table 2:** Physicochemical characteristics of cheese, during ripening.

Physicochemical Parameters	Cheese	Ripening Period (Days)					
		1	7	15	30	60	90
pH	Raw ^A^	5.41 ± 0.32 ^a^	5.29 ± 0.13 ^a^	5.34 ± 0.19 ^a^	5.25 ± 0.37 ^a^	5.39 ± 0.39 ^a^	5.35 ± 0.38 ^a^
	Pasteurized ^B^	5.51 ± 0.19 ^a^	5.25 ± 0.20 ^a^	5.24 ± 0.23 ^a^	5.46 ± 0.11 ^a^	5.43 ± 0.10 ^a^	5.49 ± 0.06 ^a^
Moisture (%)	Raw ^A^	41.73 ± 3.61 ^a^	38.89 ± 5.10 ^ac^	38.45 ± 4.77 ^ac^	38.72 ± 2.54 ^ac^	36.82 ± 5.43 ^ac^	36.36 ± 4.50 ^bc^
	Pasteurized ^A^	42.52 ± 1.75 ^a^	40.61 ± 1.30 ^ac^	38.01 ± 1.92 ^ad^	34.39 ± 4.11 ^bd^	35.30 ± 4.93 ^bd^	34.84 ± 3.88 ^bd^
Fat (%)	Raw ^A^	27.00 ± 1.84 ^a^	29.25 ± 3.98 ^a^	30.08 ± 4.09 ^a^	30.75 ± 3.43 ^a^	29.92 ± 4.48 ^a^	29.33 ± 4.20 ^a^
	Pasteurized ^B^	30.70 ± 2.25 ^a^	30.76 ± 1.83 ^a^	33.00 ± 3.02 ^a^	32.75 ± 2.19 ^a^	33.33 ± 3.06 ^a^	32.92 ± 2.92 ^a^
FDM (%)	Raw ^A^	46.35 ± 1.56 ^a^	47.79 ± 4.08 ^a^	48.71 ± 3.16 ^a^	50.10 ± 4.16 ^a^	47.18 ± 3.70 ^a^	45.99 ± 4.34 ^a^
	Pasteurized ^B^	53.53 ± 5.40 ^a^	51.80 ± 2.78 ^a^	53.17 ± 3.743 ^a^	49.92 ± 1.34 ^a^	51.04 ± 1.27 ^a^	50.46 ± 1.87 ^a^
Protein (%)	Raw ^A^	25.68 ± 2.62 ^a^	26.53 ± 1.04 ^a^	26.92 ± 0.87 ^a^	26.27 ± 1.13 ^a^	28.04 ± 1.29 ^a^	26.50 ± 1.47 ^a^
	Pasteurized ^B^	23.13 ± 1.84 ^a^	24.28 ± 2.04 ^ac^	25.77 ± 2.95 ^ac^	26.09 ± 1.12 ^ac^	26.86 ± 0.79 ^bc^	27.52 ± 2.09 ^bc^
NaCl (%)	Raw ^A^	1.22−0.24 ^ad^	1.09 ± 0.42 ^a^	1.08 ± 0.29 ^a^	1.66 ± 0.42 ^ac^	2.01 ± 0.53 ^bc^	1.80 ± 0.32 ^bcd^
	Pasteurized ^A^	1.01 ± 0.05 ^a^	1.34 ± 0.25 ^ad^	1.28 ± 0.17 ^bd^	1.64 ± 0.42 ^cd^	1.80 ± 0.41 ^cd^	1.86 ± 0.44 ^ce^
S/M (g NaCl 100 g^–1^ moisture)	Raw ^A^	2.80 ± 0.34 ^ab^	2.60 ± 0.76 ^ad^	2.71 ± 0.49 ^bd^	4.12 ± 1.10 ^c^	5.28 ± 1.65 ^c^	4.74 ± 0.99 ^c^
	Pasteurized ^A^	2.32 ± 016 ^a^	3.17 ± 0.56 ^b^	3.26 ± 0.49 ^b^	4.64 ± 1.39 ^b^	5.01 ± 1.59 ^b^	5.27 ± 1.61 ^b^
WSN/TN	Raw ^A^	5.97 ± 2.10 ^a^	6.38 ± 1.72 ^a^	7.05 ± 1.44 ^a^	5.95 ± 1.85 ^a^	5.63 ± 0.70 ^a^	6.10 ± 1.95 ^a^
	Pasteurized ^B^	4.59 ± 1.66 ^a^	5.80 ± 2.06 ^a^	5.05 ± 1.37 ^a^	4.71 ± 1.50 ^a^	4.84 ± 0.64 ^a^	5.09 ± 0.92 ^a^

Results in the same row or column for the same parameter with different superscripts (in uppercase or lowercase) are significantly different at *p* < 0.05. Capital letters A and B denote statistical different values between the raw and pasteurized cheese (considering the whole set of measurements at all times), whereas lowercase letters refer to statistically different values measured during ripening.

**Table 3:** Pearson correlation coefficients between physicochemical parameters for raw cheese.

Variable	pH	Moisture (%)	Fat (%)	FDM (%)	Protein (%)	ΝaCl(%)	S/M	WSN/TN
1. pH	-							
2. Moisture (%)	0.638 ***	-						
3. Fat (%)	−0.605 ***	−0.815 ***	-					
4. FDM (%)	−0.422 *	−0.426 *	0.871 ***	-				
5. Protein (%)	0.02	−0.019	−0.161	−0.27	-			
6. ΝaCl (%)	0.099	0.268	−0.422 *	−0.418 *	0.277	-		
7. S/M	−0.152	−0.094	−0.131	−0.269	0.276	0.93 ***	-	
8. WSN/TN	0.052	−0.174	0.228	0.194	−0.025	−0.531 **	−0.506 **	-

Pearson’s r * *p* < 0.05, ** *p* < 0.01, *** *p* < 0.001.

**Table 4:** Pearson correlation coefficients between physicochemical parameters for pasteurized cheese.

Variable	pH	Moisture (%)	Fat (%)	FDM (%)	Protein (%)	ΝaCl (%)	S/M	WSN/TN
1. pH	-							
2. Moisture (%)	0.164	-						
3. Fat (%)	−0.222	−0.701 ***	-					
4. FDM (%)	−0.118	−0.039	0.73 ***	-				
5. Protein (%)	−0.176	−0.352 *	−0.013	−0.362 *	-			
6. ΝaCl (%)	−0.313	−0.528 **	0.536 **	0.218	0.273	-		
7. S/M	−0.213	−0.779 ***	0.681 ***	0.200	0.324	0.93 ***	-	
8. WSN/TN	−0.193	0.222	0.091	0.374 *	−0.352*	−0.109	−0.166	-

Pearson’s r * *p* < 0.05, ** *p* < 0.01, *** *p* < 0.001.

**Table 5:** Fatty acid composition (%) of raw and pasteurized cheese during ripening.

Fatty Acid Ester	Cheese	Ripening Period				
		30	60	90	120	*p*-Value
C4:0	Raw	2.04 ± 0.39	2.07 ± 0.60	2.28 ± 0.29	2.31 ± 0.09	NS
	Pasteurized	2.29 ± 0.48	2.22 ± 0.35	2.34 ± 0.16	2.17 ± 0.12	NS
C6:0	Raw	1.65 ± 0.21	1.71 ± 0.30	1.80 ± 0.12	1.71 ± 0.15	NS
	Pasteurized	1.80 ± 0.19	1.77 ± 0.21	1.83 ± 0.08	1.64 ± 0.21	NS
C8:0	Raw	1.17 ± 0.12	1.20 ± 0.17	1.26 ± 0.05	1.19 ± 0.09	NS
	Pasteurized	1.23 ± 0.03	1.24 ± 0.12	1.27 ± 0.03	1.16 ± 0.17	NS
C10:0	Raw	2.98 ± 0.36	2.99 ± 0.32	3.10 ± 0.20	2.95 ± 0.20	NS
	Pasteurized	3.04 ± 0.22	3.07 ± 0.30	3.13 ± 0.15	2.86 ± 0.41	NS
C11:0	Raw	0.12 ± 0.04	0.11 ± 0.03	0.12 ± 0.02	0.10 ± 0.01	NS
	Pasteurized	0.12 ± 0.02	0.12 ± 0.03	0.13 ± 0.04	0.08 ± 0.05	NS
C12:0	Raw	3.75 ± 0.48	3.79 ± 0.29	3.84 ± 0.24	3.66 ± 0.14	NS
	Pasteurized	3.77 ± 0.41	3.82 ± 0.30	3.88 ± 0.23	3.60 ± 0.40	NS
C13:0	Raw	0.17 ± 0.04	0.17 ± 0.03	0.18 ± 0.04	0.15 ± 0.02	NS
	Pasteurized	0.18 ± 0.04	0.18 ± 0.04	0.15 ± 0.07	0.16 ± 0.02	NS
C14:0	Raw	11.61 ± 0.43	11.46 ± 0.34	11.62 ± 0.33	11.39 ± 0.52	NS
	Pasteurized	11.45 ± 0.57	11.54 ± 0.37	11.56 ± 0.38	10.87 ± 1.08	NS
C14:1	Raw	1.21 ± 0.10	1.19 ± 0.12	1.22 ± 0.18	1.10 ± 0.15	NS
	Pasteurized	1.26 ± 0.19	1.25 ± 0.20	1.24 ± 0.18	1.21 ± 0.24	NS
C15:0	Raw	1.39 ± 0.20	1.37 ± 0.19	1.38 ± 0.19	1.23 ± 0.08	NS
	Pasteurized	1.42 ± 0.20	1.39 ± 0.19	1.40 ± 0.20	1.25 ± 0.15	NS
C15:1	Raw	n.d.	n.d.	n.d.	n.d.	NS
	Pasteurized	n.d.	n.d.	n.d.	n.d.	NS
C16:0	Raw	35.23 ± 0.95 ^a^	34.93 ± 2.15 ^ab^	34.21 ± 1.16 ^ab^	32.73 ± 0.52 ^b^	*
	Pasteurized	35.06 ± 0.32 ^a^	34.46 ± 1.36 ^ab^	34.66 ± 1.49 ^ab^	33.80 ± 1.50 ^b^	*
C16:1	Raw	1.94 ± 0.18	1.92 ± 0.22	1.97 ± 0.23	1.84 ± 0.12	NS
	Pasteurized	2.00 ± 0.22	1.98 ± 0.24	1.96 ± 0.22	1.70 ± 0.55	NS
C17:0	Raw	0.63 ± 0.06	0.65 ± 0.05	0.63 ± 0.04	0.68 ± 0.09	NS
	Pasteurized	0.54 ± 0.27	0.65 ± 0.05	0.62 ± 0.07	0.66 ± 0.07	NS
C17:1	Raw	n.d.	n.d.	n.d.	n.d.	NS
	Pasteurized	n.d.	n.d.	n.d.	n.d.	NS
C18:0	Raw	8.56 ± 0.91 ^a^	8.97 ± 1.24 ^ab^	8.64 ± 1.50 ^ab^	10.28 ± 0.67 ^b^	*
	Pasteurized	8.26 ± 0.74 ^a^	8.59 ± 1.26 ^ab^	8.57 ± 1.25 ^ab^	10.01 ± 2.00 ^b^	*
C18:1 t	Raw	0.45 ± 0.07	0.43 ± 0.06	0.27 ± 0.19	0.30 ± 0.26	NS
	Pasteurized	0.42 ± 0.05	0.38 ± 0.17	0.30 ± 0.20	0.36 ± 0.24	NS
C18:1 cn9	Raw	22.21 ± 0.97	22.05 ± 0.51	22.13 ± 0.35	23.03 ± 0.43	NS
	Pasteurized	22.20 ± 0.60	22.26 ± 0.74	21.78 ± 0.98	22.60 ± 0.95	NS
C18:1 n7	Raw	0.83 ± 0.08	0.83 ± 0.04	0.86 ± 0.05	0.80 ± 0.11	NS
	Pasteurized	0.82 ± 0.02	0.86 ± 0.04	0.85 ± 0.04	0.73 ± 0.30	NS
C18:2 t	Raw	0.12 ± 0.07	0.12 ± 0.09	0.13 ± 0.09	0.10 ± 0.11	NS
	Pasteurized	0.13 ± 0.09	0.13 ± 0.09	0.13 ± 0.09	0.02 ± 0.01	NS
C18:2 c	Raw	2.45 ± 0.07 ^ab^	2.44 ± 0.03 ^ab^	2.43 ± 0.08 ^a^	2.58 ± 0.28 ^b^	*
	Pasteurized	2.49 ± 0.06 ^ab^	2.48 ± 0.09 ^ab^	2.45 ± 0.07 ^a^	2.66 ± 0.32 ^b^	*
C18:3γ	Raw	n.d.	n.d.	n.d.	n.d.	NS
	Pasteurized	n.d.	n.d.	n.d.	n.d.	NS
C18:3α	Raw	0.30 ± 0.04	0.28 ± 0.02	0.32 ± 0.05	0.23 ± 0.09	NS
	Pasteurized	0.29 ± 0.01	0.29 ± 0.03	0.32 ± 0.05	0.52 ± 0.10	NS
CLA	Raw	0.41 ± 0.07 ^ab^	0.37 ± 0.06 ^a^	0.40 ± 0.06 ^ab^	0.52 ± 0.08 ^b^	*
	Pasteurized	0.40 ± 0.07 ^ab^	0.40 ± 0.06 ^a^	0.40 ± 0.08 ^ab^	0.56 ± 0.05 ^b^	*
C20:0	Raw	0.13 ± 0.01 ^a^	0.14 ± 0.03 ^a^	0.14 ± 0.02 ^ab^	0.18 ± 0.02 ^b^	*
	Pasteurized	0.14 ± 0.02 ^a^	0.14 ± 0.02 ^a^	0.16 ± 0.04 ^ab^	0.22 ± 0.13 ^b^	*
C20:1 n9	Raw	0.07 ± 0.01 ^a^	0.09 ± 0.02 ^ab^	0.10 ± 0.03 ^b^	0.09 ± 0.03 ^ab^	*
	Pasteurized	0.05 ± 0.03 ^a^	0.09 ± 0.02 ^ab^	0.09 ± 0.02 ^b^	0.10 ± 0.05 ^ab^	*
C20:2	Raw	n.d.	n.d.	n.d.	n.d.	
	Pasteurized	n.d.	n.d.	n.d.	n.d.	
C21:0	Raw	n.d.	n.d.	n.d.	n.d.	
	Pasteurized	n.d.	n.d.	n.d.	n.d.	
C20:3 n6	Raw	0.13 ± 0.02	0.14 ± 0.03	0.14 ± 0.03	0.16 ± 0.03	NS
	Pasteurized	0.10 ± 0.06	0.12 ± 0.06	0.12 ± 0.06	0.14 ± 0.04	NS
C20:4 n6	Raw	0.22 ± 0.03	0.19 ± 0.09	0.20 ± 0.09	0.25 ± 0.01	NS
	Pasteurized	0.18 ± 0.10	0.24 ± 0.03	0.24 ± 0.06	0.24 ± 0.02	NS
C20:3 n3	Raw	n.d.	n.d.	n.d.	n.d.	
	Pasteurized	n.d.	n.d.	n.d.	n.d.	
C22:0	Raw	n.d.	n.d.	n.d.	n.d.	
	Pasteurized	n.d.	n.d.	n.d.	n.d.	
EPA	Raw	0.04 ± 0.04	0.04 ± 0.02	0.02 ± 0.02	0.04 ± 0.04	NS
	Pasteurized	0.02 ± 0.02	0.02 ± 0.02	0.04 ± 0.04	0.06 ± 0.06	NS
C22:1 n11	Raw	n.d.	n.d.	n.d.	n.d.	
	Pasteurized	n.d.	n.d.	n.d.	n.d.	
C22:1 n9	Raw	n.d.	n.d.	n.d.	n.d.	
	Pasteurized	n.d.	n.d.	n.d.	n.d.	
C22:2	Raw	n.d.	n.d.	n.d.	n.d.	
	Pasteurized	n.d.	n.d.	n.d.	n.d.	
C23:0	Raw	n.d.	n.d.	n.d.	n.d.	
	Pasteurized	n.d.	n.d.	n.d.	n.d.	
C22:4 n6	Raw	n.d.	n.d.	n.d.	n.d.	
	Pasteurized	n.d.	n.d.	n.d.	n.d.	
C24:0	Raw	n.d.	n.d.	n.d.	n.d.	
	Pasteurized	n.d.	n.d.	n.d.	n.d.	
C22:5 n3	Raw	0.04 ± 0.01	0.09 ± 0.03	0.11 ± 0.05	0.02 ± 0.01	NS
	Pasteurized	0.05 ± 0.01	0.07 ± 0.02	0.10 ± 0.04	0.15 ± 0.06	NS
C24:1	Raw	n.d.	n.d.	n.d.	n.d.	NS
	Pasteurized	n.d.	n.d.	n.d.	n.d.	NS
DHA	Raw	0.03 ± 0.01	0.06 ± 0.02	0.14 ± 0.07	0.05 ± 0.01	NS
	Pasteurized	0.23 ± 0.10	0.07 ± 0.02	0.06 ± 0.02	0.04 ± 0.01	NS
SFA	Raw	69.47 ± 0.88	69.65 ± 0.40	69.30 ± 0.17	68.67 ± 1.05	NS
	Pasteurized	69.33 ± 0.82	69.28 ± 0.69	69.78 ± 0.86	68.58 ± 1.29	NS
PUFA	Raw	3.82 ± 0.21	3.84 ± 0.09	4.06 ± 0.13	4.07 ± 0.51	NS
	Pasteurized	3.91 ± 0.53	3.90 ± 0.08	3.99 ± 0.25	4.53 ± 1.12	NS
MUFA	Raw	26.69 ± 0.75	26.48 ± 0.37	26.60 ± 0.17	27.19 ± 0.41	NS
	Pasteurized	26.75 ± 0.46	26.79 ± 0.71	26.19 ± 0.87	26.68 ± 0.50	NS
UFA	Raw	30.52 ± 0.90	30.33 ± 0.40	30.66 ± 0.14	31.26 ± 0.93	NS
	Pasteurized	30.66 ± 0.80	30.70 ± 0.68	30.19 ± 0.90	31.22 ± 0.99	NS
n-6	Raw	2.88 ± 0.11	2.94 ± 0.14	2.92 ± 0.19	3.10 ± 0.25	NS
	Pasteurized	2.88 ± 0.14 ^a^	2.98 ± 0.13 ^ab^	2.99 ± 0.14 ^ab^	3.20 ± 0.25 ^b^	*
n-3	Raw	0.40 ± 0.07 ^a^	0.50 ± 0.07 ^ab^	0.67 ± 0.20 ^b^	0.44 ± 0.06 ^ab^	*
	Pasteurized	0.58 ± 0.45	0.48 ± 0.11	0.56 ± 0.18	0.77 ± 0.20	NS
n-6/n-3	Raw	7.34 ± 1.69	6.05 ± 1.02	4.89 ± 2.15	7.19 ± 1.01	NS
	Pasteurized	6.75 ± 1.04	6.68 ± 2.00	5.79 ± 1.85	6.06 ± 1.24	NS
PUFA/SFA	Raw	0.06 ± 0.00	0.06 ± 0.00	0.06 ± 0.00	0.06 ± 0.01	NS
	Pasteurized	0.06 ± 0.00	0.06 ± 0.00	0.06 ± 0.00	0.07 ± 0.02	NS
AI	Raw	2.80 ± 0.13 ^a^	2.79 ± 0.11 ^ab^	2.76 ± 0.08 ^ab^	2.62 ± 0.03 ^b^	*
	Pasteurized	2.76 ± 0.12	2.75 ± 0.12	2.81 ± 0.17	2.60 ± 0.26	NS

Results in the same row with different superscripts are significantly different at *p* < 0.05; NS, not significant, * *p* < 0.05, n.d., not detected.

**Table 6:** Fatty acid composition (%) of milk, mature raw cheese and pasteurized cheese.

Fatty Acids	Milk	Raw Cheese	Pasteurized Cheese	*p*Value
C4:0	2.50± 0.20 ^a^	2.29 ± 0.24 ^b^	2.28 ± 0.17 ^b^	*
C6:0	1.89 ± 0.21	1.77 ± 0.13	1.76 ± 0.16	NS
C8:0	1.31 ± 0.16	1.24 ± 0.07	1.23 ± 0.11	NS
C10:0	3.30 ± 0.46	3.06 ± 0.20	3.03 ± 0.28	NS
C11:0	0.09 ± 0.05	0.11 ± 0.02	0.11 ± 0.05	NS
C12:0	4.09 ± 0.60	3.78 ± 0.22	3.78 ± 0.32	NS
C13:0	0.14 ± 0.06	0.17 ± 0.04	0.15 ± 0.06	NS
C14:0	12.20 ± 1.11 ^a^	11.55 ± 0.38 ^ab^	11.31 ± 0.75 ^b^	*
C14:1	1.25 ± 0.39	1.19 ± 0.17	1.23 ± 0.19	NS
C15:0	1.15 ± 0.39 ^a^	1.34 ± 0.17 ^b^	1.34 ± 0.20 ^b^	*
C15:1	n.d.	n.d.	n.d.	NS
C16:0	34.19 ± 2.14	33.77 ± 1.21	34.35 ± 1.48	NS
C16:1	1.76 ± 0.34	1.93 ± 0.21	1.86 ± 0.37	NS
C17:0	0.59 ± 0.09	0.65 ± 0.06	0.63 ± 0.07	NS
C17:1	n.d.	n.d.	n.d.	NS
C18:0	8.60 ± 1.85	9.13 ± 1.49	9.09 ± 1.64	NS
C18:1 t	0.42 ± 0.09 ^a^	0.28 ± 0.2 ^b^	0.32 ± 0.21 ^ab^	*
C18:1 c	21.05 ± 2.72	22.4 ± 0.56	22.08 ± 1.00	NS
C18:1 n7	0.69 ± 0.20 ^a^	0.85 ± 0.07 ^b^	0.80 ± 0.18 ^ab^	*
C18:2 t	0.18 ± 0.03 ^a^	0.12 ± 0.09 ^ab^	0.09 ± 0.02 ^b^	*
C18:2 c	2.78 ± 0.40 ^a^	2.47 ± 0.16 ^b^	2.53 ± 0.21 ^b^	*
C18:3γ	n.d.	n.d.	n.d.	*
C18:3α	0.30 ± 0.04	0.30 ± 0.11	0.40 ± 0.25	NS
CLA	0.42 ± 0.09	0.43 ± 0.14	0.46 ± 0.21	NS
C20:0	0.13 ± 0.03 ^a^	0.15 ± 0.03 ^ab^	0.18 ± 0.08 ^b^	*
C20:1 n9	0.08 ± 0.03	0.09 ± 0.04	0.1 ± 0.03	NS
C20:2	n.d.	n.d.	n.d.	NS
C21:0	n.d.	n.d.	n.d.	NS
C20:3 n6	0.18 ± 0.03 ^a^	0.15 ± 0.02 ^ab^	0.13 ± 0.07 ^b^	*
C20:4 n6	0.29 ± 0.11 ^a^	0.22 ± 0.07 ^ab^	0.24 ± 0.04 ^b^	*
20:3 n3	n.d.	n.d.	n.d.	NS
C22:0	n.d.	n.d.	n.d.	NS
EPA	0.05 ± 0.01	0.03 ± 0.01	0.05 ± 0.01	NS
22:1 n11	n.d.	n.d.	n.d.	NS
22:1 n9	n.d.	n.d.	n.d.	NS
C22:2	n.d.	n.d.	n.d.	NS
C23:0	n.d.	n.d.	n.d.	NS
C22:4 n6	n.d.	n.d.	n.d.	NS
C24:0	n.d.	n.d.	n.d.	NS
C22:5 n3	0.06 ± 0.02	0.08 ± 0.03	0.12 ± 0.03	NS
24:1	n.d.	n.d.	n.d.	NS
DHA	0.07 ± 0.02 ^ab^	0.12 ± 0.06 ^a^	0.05 ± 0.02 ^b^	*
SFA	70.24 ± 3.12	69.11 ± 0.59	69.34 ± 1.14	NS
PUFA	4.50 ± 0.46 ^a^	4.06 ± 0.26 ^b^	4.19 ± 0.69 ^b^	*
MUFA	25.26 ± 2.86	26.78 ± 0.37	26.37 ± 0.77	NS
UFA	29.76 ± 3.12	30.84 ± 0.53	30.56 ± 1.00	NS
n-6	3.55 ± 0.46 ^a^	2.98 ± 0.21 ^b^	3.07 ± 0.21 ^b^	*
n-3	0.48 ± 0.09	0.60 ± 0.2	0.63 ± 0.39	NS
n-6/n-3	7.61 ± 1.59 ^a^	5.58 ± 2.14 ^b^	5.89 ± 2.28 ^b^	*
PUFA/SFA	0.06 ± 0.01	0.06 ± 0.00	0.06 ± 0.01	NS
AI	2.97 ± 0.53	2.72 ± 0.09	2.73 ± 0.22	NS

Results in the same row with different superscripts are significantly different at *p* < 0.05; NS, not significant, * *p* < 0.05; n.d., not detected.

## Data Availability

Not applicable.
